# Strategies in Primary Care to Face the SARS-CoV-2 / COVID-19 Pandemic: An Online Survey

**DOI:** 10.3389/fmed.2021.613537

**Published:** 2021-06-02

**Authors:** Marion Eisele, Nadine Janis Pohontsch, Martin Scherer

**Affiliations:** Department of General Practice and Primary Care, Center for Psychosocial Medicine, University Medical Center Hamburg-Eppendorf, Hamburg, Germany

**Keywords:** SARS-CoV-2, COVID-19, primary health care, general practice, pandemics, Germany, health services research

## Abstract

**Background:** Primary care plays a key role in pandemics like the SARS-CoV-2 pandemic in 2020. We aimed to investigate the challenges faced and the solutions implemented in primary care.

**Methods:** One hundred and twenty-one general practitioners in Germany completed the online survey. We used open questions to examine challenges experienced and solutions implemented during the early pandemic and chose qualitative content analysis to extract and describe the meaning of the answers. We derived deductive categories from the research questions and formed inductive categories during the material reviews.

**Results:** Main challenges were: insufficient information, lack of protective equipment, need to restructure practice procedures and insufficient individual and structural pandemic preparedness, resulting in secondary challenges: fear of infection, impaired patient care, aggravated steering of patients, difficult cooperation with external entities and a not viable hygiene concept advised by authorities. Strategies to address these challenges included establishing regular team-meetings to develop new solutions, focusing on few reliable sources of information, working in alternating shifts, increasing telemedicine, establishing window and open-air practices and building networks with other health care providers. Respondents criticized the lack of consideration of their experiences in planning pandemic measures within primary care.

**Conclusions:** General practitioners successfully applied pragmatic and creative strategies in their practices during the early phase of the pandemic. Among these, communication within and between practices emerged as a key strategy. These strategies should be provided with pandemic preparedness plans. The lacking consideration of the primary care providers' experiences in planning and implementing pandemic measures needs to be addressed by stakeholders.

## Introduction

In early 2020, when SARS-CoV-2 became pandemic, three major challenges emerged: (1) the virus is highly contagious, even before the onset of symptoms (1), (2) the lack of a cure or a vaccine to prevent the spread of the disease, and (3) a blatant lack of protection material (2–4). Even well-prepared countries were unable to provide medical staff with sufficient equipment, potentially resulting in unnecessary fatalities among frontline workers (general practitioners and hospital staff) and the spread of the virus in nursing homes (5, 6). Even though the role of primary care (PC) in health emergencies is essential (7), research on pandemics revealed gaps involving primary care in pandemic preparedness and response planning. Challenges identified during the influenza A (H1N1), the SARS pandemic and previous local disease outbreaks were: Restrictions in the provision of information and guidelines, lack of personal protective equipment (PPE), performing public health tasks, obtaining support from authorities, adequate training, emotional impacts of facing an unknown pathogen, high workload, financing of epidemic/pandemic measures, organizing of practices and care of those taken ill (8, 9). In case of SARS-CoV-2, general practitioners not only had to react to a pandemic outbreak, but were also confronted with a disease without being able to follow the recommendations to protect themselves, their staff and patients from infection.

To successfully manage future pandemics, we need to understand the impact and challenges of the current pandemic on PC in various countries. We aimed to investigate the challenges faced and solutions implemented by GPs during the early SARS-CoV-2 pandemic in Germany. The research questions are: What challenges did GPs face in the early phase of the SARS-CoV-2 pandemic? How did they master these challenges? Which of these strategies could be implemented to prepare primary care for future pandemics? Which aspects go beyond the field of PC activities and need to be addressed by decision-makers?

## Materials and Methods

This paper reports on data from an online survey. GPs answered open questions on challenges and (pragmatic) solutions to handle the first months of the SARS-CoV-2-pandemic in Germany. The authors did not receive external funding for the study. No formal ethical approval procedure was carried out after the Ethics Committee of the Medical Association of Hamburg was consulted and provided a waiver as the national regulations in Germany do not require ethical approval for this kind of study (processing no. WP-044/20 from March 20 2020). The study was conducted in accordance to the Declaration of Helsinki, including, but not limited to, the guaranteed anonymity of all participants and their informed consent.

### Participant Selection and Recruitment

All GP-members of the “Listserver Primary Care” [an email discussion forum hosted by the German College of General Practitioners and Family Physicians (DEGAM); about 1,300 subscribers] were invited to participate in the online-survey on March 25th 2020. Reminders were sent out on March 27th and April 4th 2020. The survey was closed at April 7th 2020. Potential participants were informed about the URL leading to the online-survey, where they were provided with information about the purpose of the study, the voluntary nature of participation, data security and anonymity of the survey before the survey started. The completion of the survey was interpreted as informed consent to the anonymous use of the data. There was no incentive to participate.

### Survey Questions

The survey questions are provided in Table 1. The questions were written by ME based on previous research of our working group (8) and input from media reports and colleagues working on the PC frontline during the rise of the SARS-CoV-2-pandemic. The questions were pre-tested and commented on by several colleagues working in PC and (qualitative) health services research. Based on the results and comments from the pre-tests, ME (female *post-doc* psychologist) and MS (male full professor, MD, board certified in General Medicine, current president of the DEGAM) wrote the final formulation of the questions.

**Table 1:** Wording of open-ended questions in the online-survey.

	**Open ended survey questions**
1	Which situations have you experienced as challenging or difficult in your professional work during the COVID-19 epidemic?
2	What have you done specifically to meet these challenges/difficulties?
3	Which of these solution strategies worked well in your opinion and why did they work well?
4	Which of these solution strategies did not work well from your point of view and why did they not work well?
5	Which other solution strategies or measures (including pragmatic or creative ones) have you yourself or colleagues you know in other GP practices implemented to cope with the COVID-19 epidemic in your everyday work?
6	If there is anything else you would like to tell us, there is room for it here.

### Data Analysis

Following a realistic paradigm (10), we chose qualitative content analysis (11, 12) to extract and describe the meaning of the answers to the open questions (13). ME and NJP (female *post-doc* psychologist) familiarized themselves with the data. Deductive categories were derived from the research questions (challenges/solutions) and inductive categories were formed by ME and NJP during the material reviews. The newly formed categories and coding were discussed between ME and NJP in regular meetings during the process. Due to the exploratory nature of our study and to ensure that pre-existing concepts do not contaminate the interpretation of the material (12, 14), emphasis was placed on inductive category formation. To ensure intersubjective comprehensibility and credibility of the analysis (15) the results were discussed with MS and presented to colleagues working in primary health care. The data were managed with MAXqda 11 (Verbi GmbH).

## Results

Sample characteristics are provided in Table 2. The institutions mentioned in the quotes are explained in Table 3.

**Table 2:** Sample characteristics.

	***n***	**%**	**Total valid n**
Age			121
30–49	62	51	
50–65	54	45	
66 or older	5	4	
Sex			120
Female	64	53	
Male	56	46	
Position			120
Practice owner	90	74	
Employed	30	25	
Location of practice			118
Rural community/Country town (<5,000 inhabitants)	30	25	
Small town (5.000–19.999 inhabitants)	25	21	
Medium-sized town (20,000–99,999 inhabitants)	14	12	
City (100,000–999,999 inhabitants)	29	24	
Metropolis (>1 mio inhabitants)	20	17	
Number of respondents by federal state (range)	1–17	1–14	121
Total	121	100	

**Table 3:** Information on the German institutions mentioned in the quotations.

**Institution**	**German title**	**Abbreviation**	**Function**
Robert-Koch-Institute	Robert-Koch-Institut	RKI	Central institution of the Federal Government in the field of disease surveillance and prevention
Association of Statutory Health Insurance Physicians	Kassenärztliche Vereinigung	KV	Association of Statutory Health Insurance Physicians, a public corporation that organizes outpatient health care, represents the interests of doctors and is responsible for the distribution of fees for medical services
German College of General Practitioners and Family Physicians	Deutsche Gesellschaft für Allgemeinmedizin und Familienmedizin	DEGAM	Scientific association of general practitioners and family physicians
German General Practitioners Association	Deutscher Hausärzteverband	n.a.	The professional association of general practitioners. It represents the professional interests of general practitioners toward politics, health insurance companies, in medical chambers and toward the Association of Statutory Health Insurance Physicians

### Challenges

**Figure 1:**
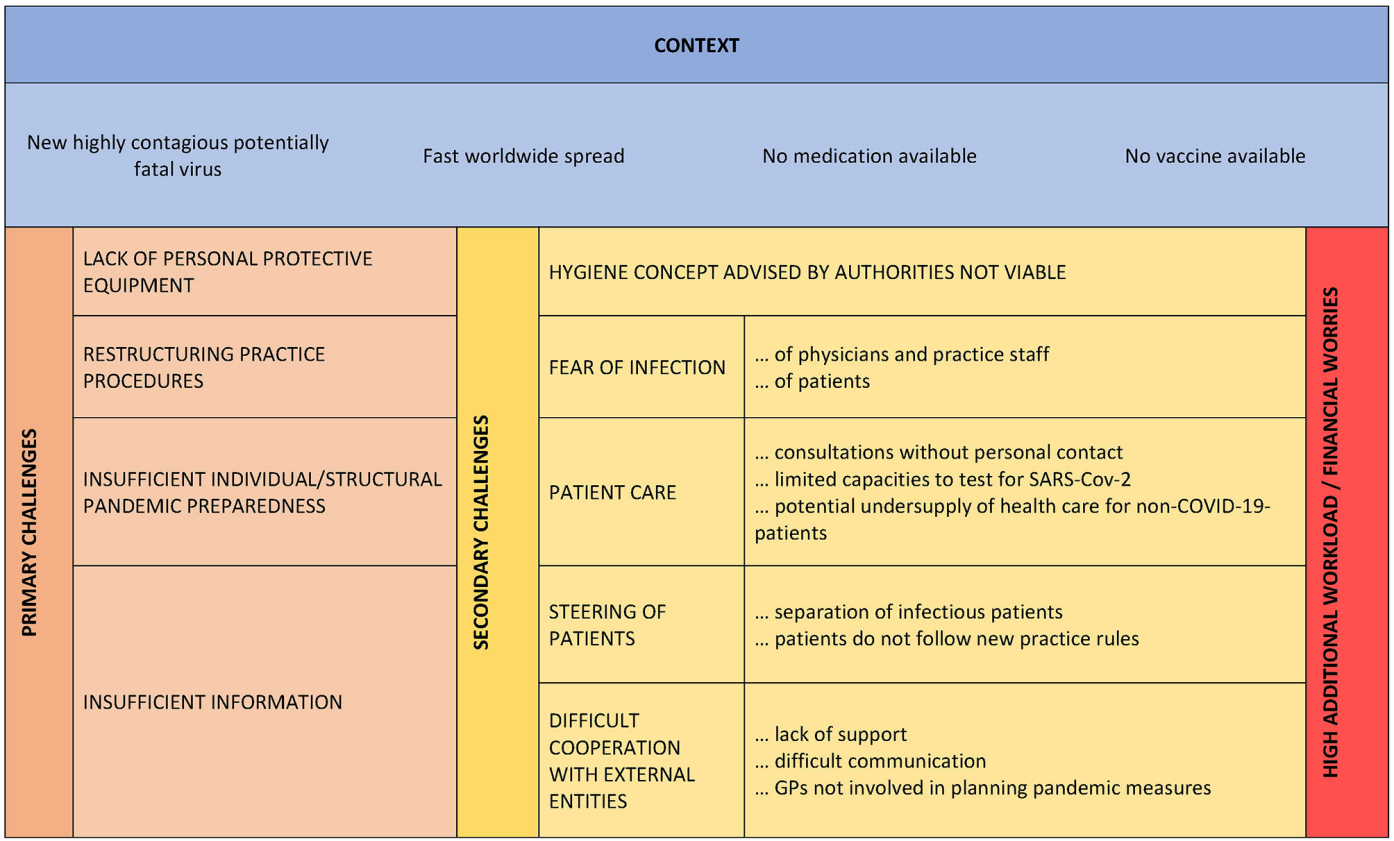
Challenges in primary care during the early phase of the SARS-CoV-2 pandemic.

The respondents reported four partly overlapping primary challenges: (1) insufficient information, (2) lack of PPE, (3) need to restructure practice procedures, and (4) insufficient individual and structural pandemic preparedness. These resulted in several secondary challenges (Figure 1).

The information for GPs was perceived as insufficient in scope, feasibility, uniformity and topicality. Even though GPs reported that good information was provided by RKI, DEGAM, General Practitioners Association and KV, they criticized that it was too much information from different sources leading to partly conflicting recommendations.

*One is overwhelmed with information (KV, General Practitioners Association, RKI, DEGAM). A common statement would be nice. (hired GP/123: 11)*

GPs reported a high daily workload to review the information and to implement corresponding measures. Mentioned information needed were: criteria for testing / quarantine, available test-centers, capacities of test-centers, emergency services and hospitals.

*Until recently, I didn't ‘dare' to send a patient, who seemed suspiciously ill, to a clinic, because I thought, that the clinics are already overflowing (yet, apparently that wasn't the case). (hired GP/389: 30)*.

The lack of sufficient PPE and disinfectant was reported to be highly challenging because — while being aware of the official instructions for hygiene — those were not viable.

*Guidelines for proceedings and for hygiene on the webpages of the RKI and the KV are not practicable. A surgical face mask must suffice for a week, for it is a scarce good (hired GP/ 303: 30)*

This resulted in the fear of own infections as well as transferring infections within the practice team and to patients while still being expected to ensure ambulant patient care.

*There is no source of supply for personal protection, on the other hand care for normal sick patients and sickened people shall be guaranteed. (practice owner/283: 11)*

This required reducing the number of personal patient consultations to a minimum, canceling routine and preventive examinations, and establishing telephone and video-consulting. Challenges were limited phone numbers and limited data volume for digital consulting. This unusual way of patient care lead to worries to oversee whether patients get worse: COVID-19 patients, patients chronically ill and other patients who were reluctant to consult a practice during the pandemic. Both — the fear of own infection (leading to practice quarantine) and the low number of patients treated—resulted in financial worries.

The need to restructure practice procedures resulted in high work load and challenges due to patients who did not obey to the new practice rules: Patients were afraid that they might not be seen by a doctor if they confess respiratory symptoms, exposure to an person with confirmed SARS-CoV-2 infection or vacation in a risk region, resulting in unprotected consultations.

*Out of fear pat[ients] occasionally mentioned possible expositions [to SARS-CoV-2 positive persons] only when seeing the doctor, after keeping it secret during triage and staying in the waiting room with other patients. (hired GP /269: 14)*

Other alarmed patients tried to urge the GPs to get them tested for SARS-Cov-2 without meeting the testing criteria.

Insufficient individual and structural pandemic preparedness lead to a sometimes helpful (e.g., test centers), but often difficult or lacking cooperation with external entities.

*No organized cooperation between clinic and outpatient clinic. Missing cooperation among GP practices and the local health authorities (hired GP/33: 11)*

Even though GPs were announced as central contact partner for patients, the GPs criticized that experts in the media were virologists and lung specialists whereas the GPs' experiences were not considered in the planning of processes and communication with the GPs was insufficient.

*Even in our district I get the feeling, that with all that has been established here (swabbing centre und fever clinics…), general practitioners have not been involved and the procedures of arranged measures were not or only poorly communicated to the GPs… (hired GP/451: 11)Policy [makers] including KV are planning without taking our experiences into account and without us… and if the official institutions (swabbing centre, hotlines, 116117 [emergency number of the Association of Statutory Health Insurance Physicians]) do not work properly, we GPs shall pull the hot chestnuts out of the fire (practice owner/353: 11)*

When seeking for help, GPs reported that it was hard or impossible to reach contact persons. Hotlines for residents were overloaded and patients were referred back to the GPs. Simultaneously politicians made promises, which could not be served by the local GPs.

*Malfunctioning test centers and meaningless promises of politicians and virologists, which we could not keep on-site. (practice owner/272: 11)*

### Strategies to Deal With the Challenges

To receive the information needed, GPs decided for few trustable and helpful information sources they checked each morning, e.g., the PC expert organization DEGAM, the federal authority for disease surveillance and prevention (Robert Koch Institute) or the Association of Statutory Health Insurance Physicians (KV). They linked up with colleagues in real or virtual networks and exchanged information and material and discussed questions. Regular telephone conferences or communication *via* messenger services groups with local ambulant and inpatient care providers were reported.

To overcome the shortness of PPE and disinfectants respondents tried ordering PPE from alternative sources like construction markets, veterinary and industrial suppliers or *via* internet. Some respondents placed a notice in their practices or an advertisement in local media to ask for donations of protection material or disinfectants. One respondent reported that he or she contacted a local brewery who was willing to produce disinfectants. Finally, when no professional masks were available anymore, the last remaining disposable masks were re-used after disinfection and GPs and medical assistants started using self-made protection material—self-made masks and face protection shields.

*Then, I had ordered a patient to build me a face shield – he has a 3D-printer. I have handcrafted a face shield for each of my employees that is wipeable and reusable and is worn additionally to the mask. (practice owner*\*403: 12)*.

Respondents reported that local practices joined forced and shared disinfectants. Other practice-networks pooled their remaining PPE and established a location for testing patients in the underground parking space of one of the practices, which was maintained by the staff of one practice supplied with the stock of PPE from all practices.

To avoid practice quarantine after unprotected consultation with a later positively tested COVID-19 patient, GPs decided to build two teams that operated separately from each other either in weekly exchange or in morning and afternoon shifts without meeting each other in between. This required good coordination *via* digital media.

*Establishment of 2 teams GP/physician assistant, who do not meet in person. Determination of handover procedures. In home office, one physician assistant oversees the call-center, one oversees the tasks in the backoffice and as practice manager. (practice owner*\*474: 12)*

To reduce the risk of infection for GPs, practice staff and patients, the GPs implemented a complete restructuring of the practice (routines): reducing personal consultations to a minimum, canceling routine controls and check-ups, policy of closed practice doors, (telephone) triage systems to decide if a patient needs to be seen in the practice, implementation of digital telephone assistants and telephone and video consultations, digital physician-patient-communication (e.g., *via* email). To assure appropriate patient care, GPs kept in close contact *via* phone with COVID-19 patients and other seriously or chronically ill patients, to avoid critical conditions and hospital admissions. There was even the idea to contact regular patients of high age and living alone, to be assured that they are doing well. Information on the measures was placed on the practice homepage, outside the door and in the practice.

*Seriously ill people get called, they do not have to make the first move to contact us. (practice owner/382: 15)*

Where disinfectant was available, disinfectant dispensers were installed at the entry. Many practices established self-made perspex panes at the registration table to reduce the risk of droplet infection and placed distance markings on the floor. Patients placed their insurance cards into the card reader by themselves instead of handing them over to the practice staff, to ensure distance among patients and between patients and practice staff. GPs reduced the number of chairs in their waiting rooms and some asked their patients to wait outdoors or allowed no more than one person in the waiting room. Practices in ground floor rooms sometimes transformed to out-of-the-window practices.

*In the next step the practice was closed, we cut a little aisle into the front garden and directed the patients via placards to a window, where they could pick up prescriptions etc. without having to enter the practice. (practice owner/403: 12)*

Patients with signs of respiratory infections were separated from other patients by: special consultation hours with strict time frames for each consultation to avoid the patients meeting in the practice, establishing consulting rooms for patients with respiratory infections only and foregoing stops in the waiting room. Where no separate room was available, a room in the cellar was converted into a consulting room, an external room was rented or patients with respiratory infections were examined and tested outside the practice: In the car park, the back yard or within a provisional hut in front of the practice. Patients were also guided on how to carry out a throat and nasal swab by themselves if the practice lacked PPE.

*Mobile rolling surgery table with all the material, swabs and examinations take place outdoors. The patient stays behind a 2m-distance marker, we are only approaching him/her for swabs and/or examinations. (practice owner/186: 12)*.

Following the restructuring of the practice procedures, the patients needed to be accustomed to the new practice procedures and it was important to provide a maximum of transparency on why the measures were taken.

“*…waiting INFRONT of the practice (…) causes more or less chaos depending on pat[ients'] mindset, because for some people its seems to evoke the feeling as if there was a big rush and they could come off badly (…) (hired GP/451: 14)*.

The best way to handle challenging patients was to establish clear rules (e.g., criteria for getting tested) and to communicate the same rules by all members of the practice staff. This required daily team meetings, where the current situation and necessary changes in practice procedures were discussed and defined and each member of the practice team had the possibility to contribute own ideas.

*With a time lag, all (…) measures were necessarily accepted, due to stringent proceeding without exceptions. Prerequisite: Daily meetings with staff prior to every shift, no matter if 100 patients are already waiting impatiently!!! (practice owner/166: 13)*.

The team-meetings were also used to share worries and find unusual solutions: One respondent reported, that the GP of the team supported the physician assistants in taking up patients' phone calls, because the same information was better accepted when told by the GP than by physician assistants. One GP reported that his/her son ran errands like bringing the prescription to the pharmacy and the medication to the patient, during the time the schools were closed. In one practice, the physician assistants cleaned the practice, because the charwomen was at high-risk for severe a course of COVID-19. A strong team spirit positively affected patients and co-workers.

‘*Complete package' is working and accepted, because employees, patients and nursing services have got the feeling that the practice has a plan and cares for their concerns. (practice owner/353: 13)*

The lack of sufficient pandemic preparedness could be partly compensated. An important factor was networking. Given a well-established practice network, practices were able to establish their own test-centers to save PPE. New networks were established to compensate a lack of information flow between ambulant and inpatient structures and collaboration with nursing homes were strengthened.

*Establishment of a WhatsApp group in county [with] all resident doctors, hospital doctors (practice owner/87: 12)Am developing a plan with a nursing home for palliative care/care for affected residents, who do not want the intensive care treatment any longer (practice owner/353: 30)*

Nonetheless, the failure of not considering the GPs experiences in planning the pandemic measures could not be fully compensated during the first phase of the pandemic and resulted in the following claim:

*[A] representation of general practice in committees that deal with pandemic planning, [is] urgently needed. (hired GP/269: 30)*

## Discussion

We identified four major challenges that GPs subjectively faced in the early stages of the SARS-CoV-2-pandemic in Germany in early 2020. Those were partly interlinked: lack of information, PPE and individual and structural pandemic preparedness as well as the need for a comprehensive restructuring of practice procedures. GPs reported on many strategies to obtain sufficient information (e.g., concentrating on few reliable sources and creating or using already existing networks for information exchange), to overcome the shortage of PPE (e.g., tapping new sources for PPE and disinfectant or reusing disposable equipment), restructuring practice procedures and use of additional premises (e.g., working in alternating shifts, increasing telemedicine and establishing window and outdoor practices) and compensating for the lack of readiness (e.g., by networking with other health care providers).

### Strengths and Limitations

This study sheds light to the adaptation of primary care to an emergent situation. Besides the challenges this study focuses on the solutions found and therefore gives valuable information and inspiration on how to creatively solve challenges in an exceptional situation. There are some limitations that need to be considered. The study was conducted in Germany and generalizability to other settings might be limited. However, the solutions provided can be adopted to other settings or at least inspire PC providers on how to find solutions to the challenges of a pandemic. Recruiting the users of an established email forum do not necessarily represent the German GPs, but rather those who are more interested in DEGAM, guidelines and perhaps even digital technologies. However, male and female GPs from all federal states of Germany, of all age groups, being either self-employed or employed took part in our survey. We therefore assume that we draw a comprehensive picture of the challenges and solutions in Germany.

### Comparison With Existing Literature

**Figure 2:**
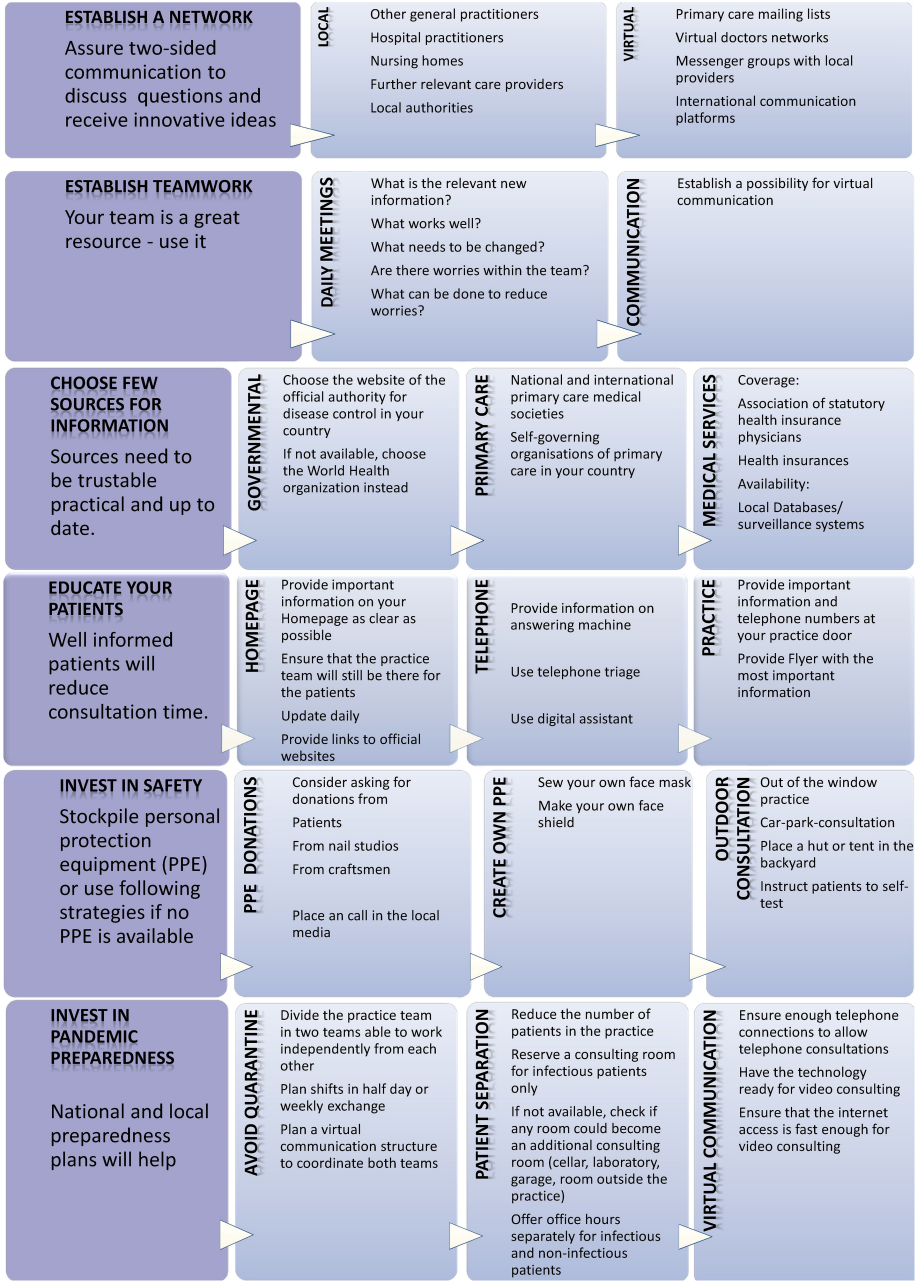
Strategies to face a pandemic in primary care.

**Figure 3:**
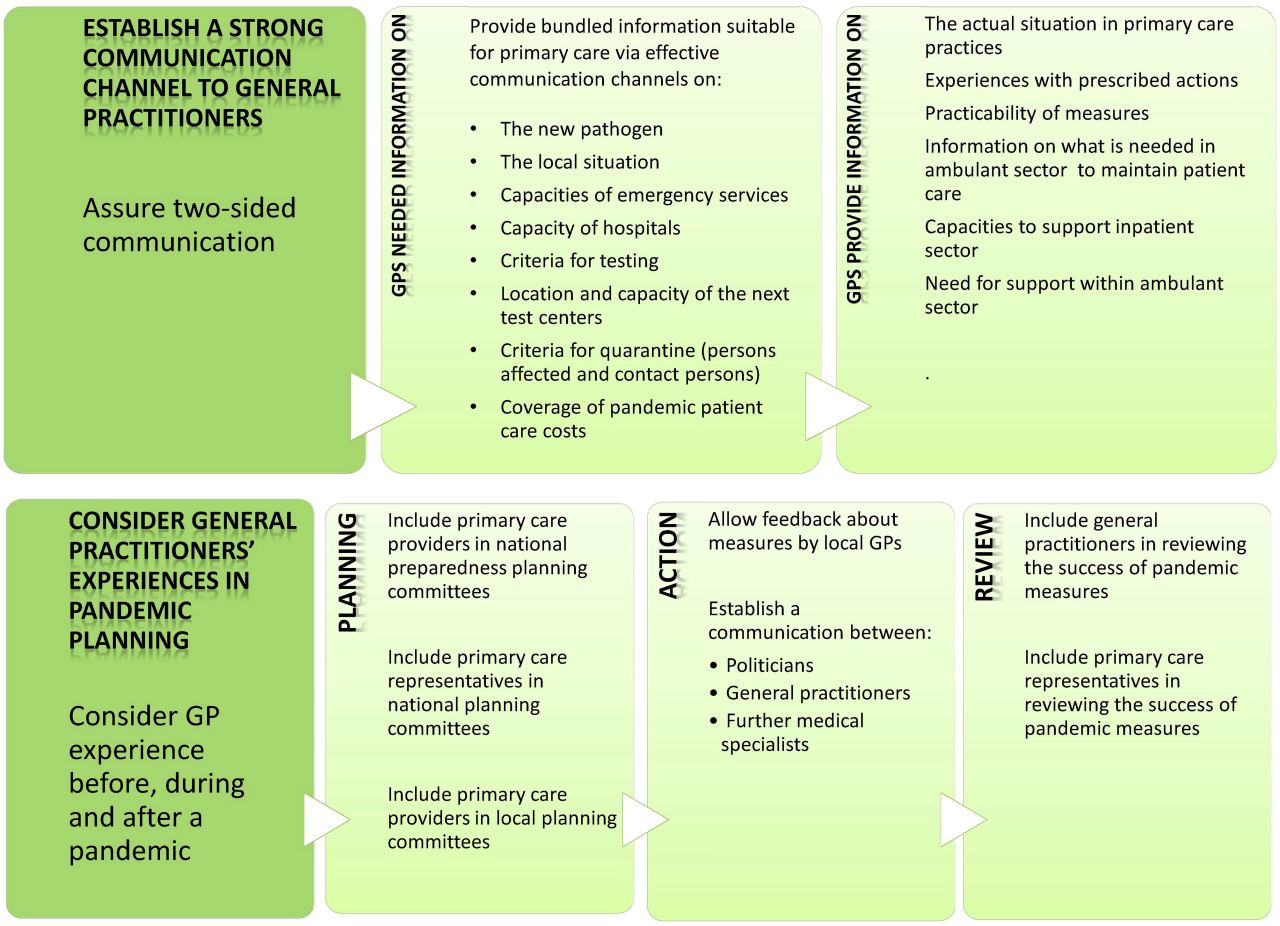
Strategies to face a pandemic—stakeholder options.

From the experiences of the respondents in this study, key strategies for PC providers and stakeholders in the early stages of a pandemic in ambulatory PC structures were derived and supplemented by earlier research results (Figure 2: for PC providers; Figure 3: for stakeholders). The combination of both will help to improve future pandemic preparedness in primary care.

The four primary challenges confirm earlier studies conducted in PC. The need for *better information and communication* with public health actors has been identified in reports from the COVID-19 frontline (2) and earlier research (9, 16). The information should be bundled and tailored to PC by a central office and communicated *via* effective channels. Then each GP could invest the time saved in patient care, where it is needed. However, in later phases of the pandemic the German College of General Practitioners and Family Physicians provided a living, weekly updated guideline on SARS-CoV-2 in primary care (17) and joined forces with the General Practitioners Association. A joint crisis team met weekly and provided all members with information letters and an initially daily DEGAM podcast was set up to inform GPs (18).

In the study of Kunin et al., GPs also reported that a channel for concerns and feedback to the authorities must be created when implementing pandemic measures (19). Even though position papers of the DEGAM were published to communicate with policy makers (20), there is no direct communication channel in Germany. A two-way communication channel between the PC frontline and the stakeholders is necessary to optimize a pandemic response.

The global *shortage of PPE* during the early SARS-CoV-2 pandemic was a worldwide major challenge (6, 21–23). Hygiene concepts released did not consider this, so the GPs improvised, treated the patients outdoors and offered self-swabbing—its feasibility has already been demonstrated (24).

GPs invested great efforts in the *restructuring of practice procedures* in order to protect practice staff from infections, which has also been reported by Flemish GPs (23). Telephone- and video-consulting was implemented to maintain patient care. Recently a guide to telephone and video consulting for COVID-19 patients was published (25). Routine consultations were cancelled and telephone triage has been introduced before patients are admitted to the practice. This led to the challenge to steer indignant and unsettled patients who felt threatened by the pandemic and entered the practice or kept their symptoms secret, which was already found at the 2009/A/H1N1 pandemic (26). The familiarity between GP and patient is an important aspect that differs from other medical specialists. Therefore, patients need to understand why the changes in practice are established and that they are not left alone.

The *insufficient individual and structural preparedness* already became apparent in the above aspects. In addition, financial and political aspects stood out. While GPs in the U.S. were confronted with a lack of reimbursement of telehealth care and Senate's stimulus package of March 2020 does not provide funding for self-employed GPs (27), financial worries, especially due to quarantine, are unnecessary for GPs in Germany. Section 56 of the infection protection law stipulates that GPs and their practice staff are entitled to compensation if the practice is banned for infection protection reasons (28). The concerns are subjected to the lack of information. Less clearly regulated, is the loss of income for the time of reducing patient contacts to a minimum. As long as financial issues are settled *post-hoc*, the financial worries of PC providers will remain during the pandemic. Internationally, there are good examples of *ad-hoc* support: Australia provided incentive payments to keep practices open, transferred personal billing practices to telehealth consultations and offered financial support during the crisis (29). New Zealand reimbursed GPs for the costs of the transition to telehealth care (2).

GPs reported difficulties in working with external institutions. They lacked information on hospital capacities for critically ill patients. Later during the pandemic, a monitoring system for intensive care beds (with ventilation options) was set up in Germany. Further key aspects were overloaded hotlines and the unavailability of health departments and politicians who made promises that could not be met by the GPs. This reflected that PC representatives were only marginally involved in development of pandemic preparedness plans and measures. A claim to include PC physicians in the pandemic planning was already made by Dunlop et al. (30). Clark went even further: “This need goes beyond having representatives of physician specialty organizations; rather, there should be a mechanism to solicit input from providers currently practicing in PC (not hospital-based) settings” (31). Our findings suggest that there is an urgent need to establish a strong two-way communication channel between care providers and politicians, because no one can better provide information on the actual situation at the frontline than the frontline workers themselves. This cooperation provides the chance to exploit the maximum potential of options to handle a health emergency. The time to establish the communication channels is now—during the ongoing pandemic. Furthermore, these communication channels should be implemented permanently, as they have the potential to strengthen the overall functioning and resilience of the PC system.

### Implications for Research and/or Practice

The implications for practice are presented in Figures 2 and 3. Despite various challenges, GPs have successfully applied pragmatic and creative strategies in their practices to overcome the difficulties during the early SARS-CoV-2 pandemic. These valuable strategies presented here should be considered in primary care and preserved and provided with future pandemic preparedness plans. Increased communication within and between practices to share knowledge and experiences proved to be a key strategy. It allowed to deal with worries and establish innovative ideas such as sharing forces and pooling remaining PPE. Pandemic preparedness plans should include options to ensure this kind of communication. Stakeholders should address the reported system deficits such as insufficient flow of information and lack of consideration of the experience of GPs in the planning and implementation of pandemic measures. As a first step, stakeholders must recognize that (1) the key medical specialists who will provide patient care during a pandemic are the general practitioners and (2) that their practical experiences provide a valuable resource to support the feasibility of pandemic measures to ensure patient care.

## Data Availability Statement

The datasets presented in this article are not readily available because of the assurance to the participants that the full raw data would not be shared publicly, and that all attempts would be made to maintain confidentiality. Respondents did not agree to the sharing of the full raw data. Requests to access the datasets should be directed to m.eisele@uke.de.

## Ethics Statement

Ethical review and approval was not required for the study on human participants in accordance with the local legislation and institutional requirements. Participants were notified before participation that their informed consent would be assumed if they voluntarily completed the online survey.

## Author Contributions

ME and MS planned the study and developed the survey questionnaire. ME and NP analyzed the data and drafted the manuscript. MS provided advice at different stages of the research process and manuscript drafting. All authors had access to the complete data and approved the final version of the manuscript.

## Conflict of Interest

ME and NP are members of the German College of General Practitioners and Family Physicians (DEGAM). MS reports personal fees from A+ Videoclinic GmbH, outside the submitted work and is president of the DEGAM.
